# Albumin and surgical site infection risk in orthopaedics: a meta-analysis

**DOI:** 10.1186/s12893-016-0186-6

**Published:** 2017-01-16

**Authors:** Peizhi Yuwen, Wei Chen, Hongzhi Lv, Chen Feng, Yansen Li, Tao Zhang, Pan Hu, Jialiang Guo, Ye Tian, Lei Liu, Jiayuan Sun, Yingze Zhang

**Affiliations:** Department of Orthopedic Surgery, the Third Hospital of Hebei Medical University, No. 139 Ziqiang Road, Qiaoxi District, Shijiazhuang, 050051 People’s Republic of China

## Abstract

**Backgroud:**

Surigical site infection has been a challenge for surgeons for many years, the prevalence of serum albumin <3.5g/dL has been reported to be associated with increased orthopaedic complications. However, the prognostic implications and significance of serum albumin <3.5g/dL after orthopaedic surgeries remain ambiguity. In this study, we performed a meta-analysis to access the predictive value of serum albumin level on SSI.

**Methods:**

A basic data search was performed in PubMed and Web of Science, in addition, references were manually searched. All of the observational studies contained preoperative albumin, outcomes of SSI or valuable data that could be abstracted and analysed for meta-analysis in orthopaedics. All of the studies were assessed using the classic Newcastle Ottawa Scale (NOS). They conformed to critical quality evaluation standards, and the final data analysis was performed with RevMan 5.2 software.

**Results:**

A total of 112,183 patients included in 13 studies were involved. The pooled MD of albumin between the infection group and the non-infection group was MD = −2.28 (95 % CI −3.97–0.58), which was statistically significant (*z* = 2.63, *P* = 0.008). The pooled RR of infection when comparing albumin <3.5 with albumin >3.5 was 2.39 (95 % CI 1.57 3.64), which was statistically significant (*z* = 4.06, *P* < 0.0001). Heterogeneity were found in the pooled MD of albumin and in the pooled RR for infection (*P* = 0.05, I^2^ = 61 % and *P* = 0.003, I^2^ = 68 %). No publication bias occurred based on two basically symmetrical funnel plots.

**Conclusion:**

Our meta-analysis demonstrated that an albumin level <3.5 g/dL had an almost 2.5 fold increased risk of SSI in orthopaedics, although this conclusion requires well-designed prospective cohort studies to be confirmed further.

## Background

The most common complication an orthopaedics patient can confront is surgical site infection (SSI). SSI has been a challenge for surgeons for many years, and the trends currently prefer the development of post-operation management to decrease the SSI rate. However, can SSI be predicted using less invasive or more tolerable tests? Recently, researchers have shown that malnutrition has links with serious complications in orthopaedics, but as many as 50 % of cases of pre-existing malnutrition are unrecognized in the hospital population [1, 2], with a reasonable explanation being that observable signs of malnutrition appear only in extreme cases. Because serum albumin has high sensitivity, it also can be used to determine and screen for nutritional status [2, 3]. Owoicho Adogwa et al. suggested that preoperative low albumin (serum albumin <3.5 g/dL) was an independent risk factor for postoperative SSI in spine fusion [4], Jason D. Walls et al. identified 49,475 total hip arthroplasty (THA) patients and found that low albumin was a significant risk factor for increased mortality and major morbidity in THA [5]. However, controversy exists as well, with Jiong Jiong Guo finding that ALB had only a weak relationship with delayed wound healing after hip fractures in the elderly [6]. M. Hedström demonstrated that preoperative serum albumin could not be used to predict postoperative deep wound infection [7]. Identical results regarding the relationship between low albumin and SSI from large systematic reviews have not yet been obtained; therefore, we aim to perform a meta-analysis to investigate whether low albumin is effective in predicting SSI and to estimate the relative infection rates in patients with normal albumin and low albumin.

## Methods

### Literature search

An online search was performed in two databases (Web of Science, PubMed) from 1970 to 2015. Selected references were manually reviewed. The main search terms were “albumin”, “risk” and “infection”. The search details in PubMed were as follows: (Etiology/Broad[filter]) and (“albumin”[tiab]) or (“hypoalbuminemia”[tiab]) and “infection”[tiab] and ((“spine”[tiab] or “hip”[tiab] or “knee”[tiab] or “shoulder” [tiab] or “joint” [tiab] or “fracture” [tiab] or “arthroplasty” [tiab] or “orthopaedics”[tiab]). In Web of Science, the details were as follows: (Ts = albumin or Ts = (hypoalbuminemia)) and Ts = infection and Ts = risk, refined by orthopaedics.

### Exclusion and eligibility criteria

Studies were required to meet the following eligibility criteria: 1) studies regarding surgical site infections (SSI, superficial SSI, deep SSI, organ space SSI) in orthopaedics; 2) studies including infection and non-infection groups subdivided by serum albumin <3.5 g/dL and serum albumin >3.5 g/dL; 3) studies with sample sizes with a mean ± standard deviation of albumin between an infection group and a non-infection group; 4) cross-sectional studies, cohort studies, and cross-sectional, cohort studies containing assessable data or reported risk ration (RR) with 95 % confidence interval (CI) of infection risk between two groups or other cross-sectional studies, cohort studies, and cross-sectional, cohort studies containing assessable data; and 5) studies providing sufficient data to fulfil the contingency tables.

Non-English-language articles, case reports, reviews, duplicate papers with same results, and conference reports were excluded. Original articles without control groups were excluded, and articles with incomplete or unacceptable information were excluded.

### Data collection

Standardized two-by-two contingency tables were used to record the following abstracted data: title, first author, country, publication year, research year, study type, average age of subjects, surgery type, patient number and the mean ± standard deviation of albumin in infection and non-infection groups; Relevant variables were carefully read and extracted from each study. Missing data were supplemented by contacting the corresponding authors.

All of the studies were assessed for quality evaluation standards using the classic Newcastle Ottawa Scale (NOS) [8].

### Statistical analysis

The Cochrane Collaboration’s RevMan 5.2 software was used for the data analysis. Pooled mean difference (MD) with 95 % CIs for continuous variables and enumeration data for Pooled RR with 95 % CIs were calculated, and the Z test was performed to determine overall effects. If the heterogeneity between studies was statistically significant (I^2^ > 50 %), a random effects model was used for further sensitivity analysis; otherwise, a fixed effects model was used (I^2^ < 50 %).

## Results

### Results of literature search and evaluation of methodological quality

**Fig. 1:**
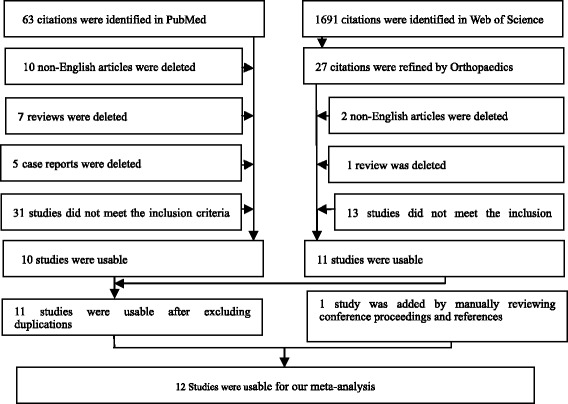
Flow diagram showing selection of studies

Two authors (Yuwen Peizhi and Chenwei) sorted and reviewed all of the titles and abstracts of the retrieved articles; 25 studies finally met the eligibility criteria. Each full text article was read by two reviewers, and 12 studies involving 112,183 patients were ultimately placed on the short list to complete the form. The quality of the articles was assessed according to the literature quality evaluation criteria (Fig. 1, Tables 1 and 2).

**Table 1:** Characteristics of selected studies for dichotomous variable meta-analysis

First author, Year	Country	Study design	Age (year)	Year of operation	Time of Infection	Type of surgery	Type of infection	Infection group		No infection group		NOS
								albumin <3.5	albumin >3.5	albumin <3.5	albumin >3.5	
DM Masatu, 2010 [16]	Tanzania	Cohort	36.11 ± 14.38	3–12,2009	30 days	femoral fractures	SSI	7	2	18	73	7
Lan B.MC Phee, 1998 [9]	Australia	Cohort	infection 54 ± 13, primary 53 ± 17	11/1984–5/1995	not mentioned	spine metastases	SSI	9	5	47	9	6
Owoicho Adogwa, 2014 [4]	US	Cohort	53.8 ± 17.0	2011–2013	12 months	spine fusion surgery	deep	2	2	55	77	
							superficial	1	0	56	79	7
Charles L Nelson, 2015 [12]	US	Cohort	not mentioned	2006–2013	during hospitalization	TKA	superficial	20	228	1546	35,298	8
							deep	6	42	1560	35,484	8
							Organ space	7	54	1559	35,472	8
Carlos J. Lavernia, 1999 [11]	US	Cohort	64.6 ± 15.62	1/-1-31/12, 1997	during hospitalization	THA and TKA	deep	1	2	21	95	7
Klein, Jeffrey D, 1996 [15]	US	Cohort	45	1990–1992	3 Year	lumber decompression and fusion	SSI	9	2	20	83	7
Jason D. Walls, 2015 [5]	US	Cohort	not mentioned	2006–2013	30 days	THA	superficial	24	164	1098	22,952	7
							deep	8	62	1114	23,054	7
							Organ space	3	46	1119	23,070	7
Hiroyuki Hayashi, 2015 [19]	Japan	Cohort	53.8	4/2006–6/2013	not mentioned	spondylectomy	SSI	2	6	16	101	8
Daniel D. Bohl, 2015 [18]	US	Cohort	not mentioned	2011–2013	30 days	THA and TKA	SSI	45	457	1919	47,182	8

**Table 2:** Characteristics of selected studies for continuous variable meta-analysis

First author, Year	Country	Study design	Age (year)	Year of operation	Time of infection	Type of surgery	Infection group		No infection group		NOS
							N	albumin	N	higher albumin	
Lena Gunningberg, 2008 [20]	Sweden	Cohort	66.6 ± 10.1	9/2004–4/2005	30 days	Orthopaedic surgery Thoracic surgery	6	39.5 ± 1.0	88	42.2 ± 4.0	8
M. Hedstrom1, 1998 [7]	Sweden	Cohort	infection:82 ± 11; non-infection 81 ± 9	1993–1994	30 days	femoral neck fractures with two Olmed cancellous bone screws	13	38 ± 5	415	37 ± 4	7
George N. Guild MD, 2012 [17]	US	Cohort	not mentioned	1/2001–5/2007	30 days	orthopaedic trauma surgery	15	31.6 ± 3.6	49	35.2 ± 3.8	6
Lan B.MC Phee, 1998 [9]	Australia	Cohort	infection 54 ± 13, primary 53 ± 17	1/1984–5/1995	not mentioned	spine metastases	14	36.5 ± 5.2	56	39.7 ± 4.8	6

### Main meta-analysis

#### Albumin difference between the infection and non-infection groups

**Fig. 2:**
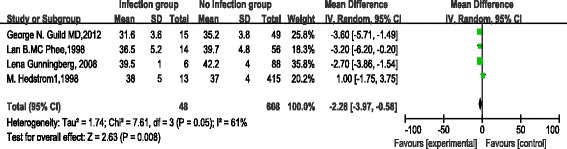
Forest plot of pooled albumin MD between albumin <3.5 mg/dL group and albumin ≥3.5 mg/dL group

Four studies reported available albumin data with relative higher heterogeneity (*P* = 0.05, I^2^ = 61 %). A random effects model was applied for meta-analysis, and the results showed that preoperative albumin was significantly lower in the infection group than in the non-infection group (OR = −2.28, 95 % CI [−3.97, −0.58], *P* =0.008) (Fig. 2).

#### SSI rate between the infection and non-infection groups

**Fig. 3:**
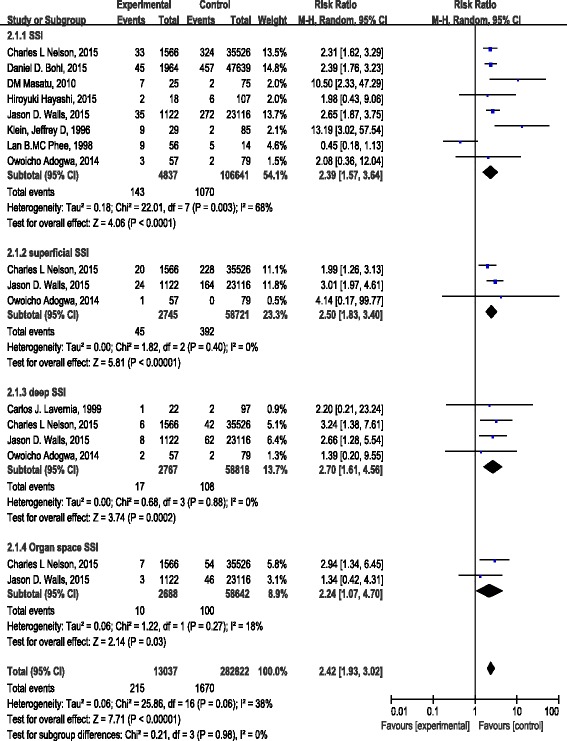
Forest plot of pooled OR of infection rate in albumin <3.5 mg/dL and albumin ≥3.5 mg/dL

Nine studies (Lan B. MC Phee contain both Albumin difference and SSI rate) reported the incidence of SSI in both groups. In SSI group, the infection rate was 2.96 % (143/4837) in the albumin <3.5 g/dL group and 1.00 % (1070/106,641) in the albumin >3.5 g/dL group, (RR = 2.39, 95 % CI [1.57 3.64], which was statistically significant (*Z* = 4.06, *p* < 0.0001) in a random model (I^2^ = 68 %). In superficial SSI subgroup, the infection rate was 1.64 % (45/2745) in the albumin <3.5 g/dL group and 0.67 % (392/58,721) in the albumin >3.5 g/dL group, (RR = 2.46, 95 % CI [1.81 3.35], *Z* = 5.73, *p* < 0.00001 in a fixed model (I^2^ = 0 %). In deep SSI subgroup, the infection rate was 0.61 % (17/2767) in the albumin <3.5 g/dL group and 0.18 % (108/58,818) in the albumin >3.5 g/dL group, (RR = 2.62, 95 % CI [1.56 4.42], *Z* = 3.62, *p* = 0.0003) in a fixed model (I^2^ = 0 %). In organ space SSI subgroup, the infection rate was 0.37 % (10/2688) in the albumin <3.5 g/dL group and 0.17 % (100/58,642) in the albumin >3.5 g/dL group, (RR = 2.17, 95 % CI [1.13 4.15], *Z* = 2.34, *p* = 0.02 in a fixed model (I^2^ = 18 %) (Fig. 3).

#### Sensitivity analysis

Regarding the pooled MD of albumin between the infection group and the non-infection group was MD = −2.28 (95 % CI −3.97–0.58), which was statistically significant (*z* = 2.63, *P* = 0.008). Regarding the overall effect RR (95 % CI) of the difference in albumin, the SSI rates between the compared groups in a random model were 2.39 (95 % CI 1.57, 3.64) (*z* = 4.06, *P* < 0.001), superficial SSI, deep SSI and organ space SSI between the compared groups in the fixed model were 2.46 (95 % CI 1.81, 3.35), 2.62 (95 % CI 1.56, 4.42) and 2.17 (95 % CI 1.13, 4.15), respectively. All showed statistically significant (*z* = 5.73, *P* < 0.00001; z =3.62, *P* = 0.0003 and z =2.34, *P* = 0.02, respectively), the results were consistent between the random and fixed effects models, suggesting that all of the findings in our study were fundamentally reliable (Figs. 2 and 3).

#### Publication bias

**Fig. 4:**
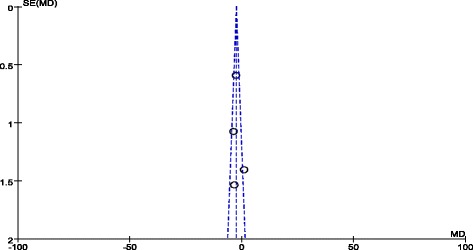
Funnel plot for publication bias. The symmetrical panel suggested no publication bias for albumin MD meta-analysis

**Fig. 5:**
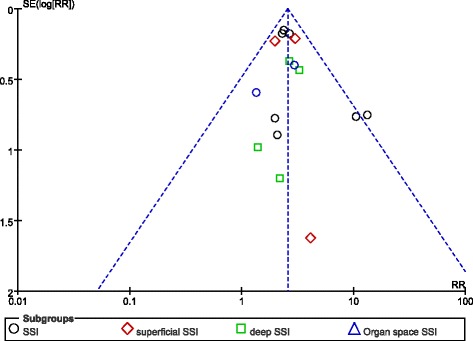
Funnel plot for publication bias. The symmetrical panel suggested no publication bias for infection rate meta-analysis

The funnel plots of pooled MD in albumin levels between the infection and non-infection groups and in the incidence of SSI in the two groups were both basically symmetrical, demonstrating no significant publication bias (Figs. 4 and 5).

## Discussion

The meta-analysis indicated that an albumin <3.5 mg/dL had an almost 2.5fold increased risk of SSI in orthopaedics, and these outcomes were statistically significant (*p* < 0.05) and robust. Many factors have been indicated and proved to have effects on SSI; among these factors, malnutrition has stood out, and a broad array of serological laboratory values, such as a serum albumin <3.5 mg/dL, have presented a significantly increased risk of infection in spine metastases [9], spine fusion [4], joint arthroplasty [10] and hip fracture [5, 11]. Theoretically, our wound healing progress was fundamentally based on our own knowledge of the potential relationship between nutrition and SSI, which could help us forecast SSI or even through some potent treatment, maintain the patient’s nutritional status, which in turn could promote the body’s resistance to pathogenic bacteria, obtaining satisfactory clinical results.

Charles LN et al. reported that low serum albumin had a more dominant association with complications after TKA than obesity [12]. Carlos J. L et al. evaluated the standard preoperative laboratory tests of 119 patients and demonstrated that preoperative nutritional status was an excellent predictor as SSI, as well as controllable factors for postoperative complications in patients undergoing joint replacement surgery [11]. Dickhaut et al. showed that low serum albumin and a low lymphocyte count increased the risk of wound complications in ankle amputations [13]. A shoulder arthroplasty study referred to a general prevalence of malnutrition of 7.6 %, and TSA patients with a preoperative albumin <3.5 g/dL tended to experience greater morbidity after surgery than patients with albumin in the normal reference ranges [10].

We perceived some heterogeneity between the included studies, especially in the infection rate comparison. The most dominant manuscript contributing to the heterogeneity of SSI incidence was Lan B. MC Phee et al. (1998) [9], after removing this study from consideration, the heterogeneity became relatively lower (*P* = 0.18, I^2^ = 33 %), as determined by a fixed effects model. Other possible reasons for heterogeneity were that low albumin was not the only susceptibility factor for SSI, obesity, age, low total lymphocyte counts, transferrin and combinations of these factors could all exerted an impact on SSI [14], and there were inconsistent factors among these studies. The sources of heterogeneity of pooled MD with regard albumin consisted mainly of the study by M. Hedström1 (1998), which provided only medians and interquartile ranges of albumin. After removing this study, the adjusted heterogeneity was *P* = 0.75 (I^2^ = 0 %), as determined by a fixed referenced model, indicating very acceptable, low heterogeneity.

Despite the existing heterogeneity, we still found positive findings that low albumin was related to SSI and that albumin <3.5 g/dL could be seen as a risk factor for SSI in orthopaedics.

In our study, the incidence of SSI in orthopaedics of SSI comparison was 1.09 % (1213/111,478), the rate of superficial SSI was 6.8 % (4371/64,466), the rate of deep SSI was 0.20 % (125/61,585) and the rate of organ space SSI was 0.18 % (110/61,330). Incidence of SSI in low albumin group in each comparison were higher than the normal albumin group [4, 5, 7, 9, 11, 12, 15–20]. Approximately 40 % of admitted adult patients were undernourished, in particular, 4.3 % of community-dwelling adults were in the same situation [21–23]. Ozkalkanli MY also referred to similar rates of malnutrition and morbidity in orthopaedic surgery of 3.5 and 4.1 %, respectively [23]. Normally, SSI rates are generally higher in orthopaedics patients than in other types of surgeries due to various and serious types of trauma [16, 24]. A rate of SSI of 7.1 % in spine surgery was reported by Satoru Demura [25]. In spinal metastases, the rate of SSI has been reported to range from 6.8 to 20 % [18, 19, 26, 27]. Huang demonstrated higher acute infection rate in THA patients with low albumin [28]. A systematic review indicated the incidence of SSI after total hip arthroplasty ranged from 0.2 % before discharge to 1.1 % for the period up to and including 5 years postoperation [29]. While in TKA, the rates of superficial and prosthetic joint infections were 2.9 and 0.80 %, respectively [30]. These findings identified our consequences, also indicating the important and severe current situation in orthopaedics. Given that malnutrition contributes to inadequate and incomplete wound healing, it could also lead to more devastating outcomes; parameters such as serum albumin and TLC are easily obtained, stable, inexpensive and established biochemical markers of nutritional status [31–33]. Therefore, we recommend thorough nutritional consultation for each hospitalized patient in orthopaedics.

There were several limitations to our meta-analysis. Firstly, heterogeneity existed due to small search range, relatively low-quality and fixed literature types. Secondly, information on potential confounding factors such as age, general health and co-morbidities were lacking for different aspects of analysis in included articles. Thirdly, SSI got many combined influencing factors even though low albumin can dramatically affects it [1, 3, 10, 13, 20, 21], interference between those factors was not identified.

## Conclusion

Our meta-analysis found that albumin <3.5 g/dL had an almost 2.5 fold increased risk of SSI in orthopaedics. Prospective, multicentre studies should be performed to verify this conclusion.

## Abbreviations

CI: Confidence interval
MD: Mean difference
NOS: Newcastle Ottawa Scale
RR: Relative risk
SD: Standard deviation
SSI: Surgical site infection
THA: Total hip arthroplasty
TKA: Total knee arthroplasty
TSA: Total shoulder arthroplasty
